# Evolution of KIPPIS as a versatile platform for evaluating intracellularly functional peptide aptamers

**DOI:** 10.1038/s41598-021-91287-z

**Published:** 2021-06-03

**Authors:** Daiki Kashima, Masahiro Kawahara

**Affiliations:** 1 Department of Chemistry and Biotechnology, Graduate School of Engineering, The University of Tokyo, 7-3-1 Hongo, Bunkyo‐ku, Tokyo, 113-8656 Japan; 2 Laboratory of Cell Vaccine, Center for Vaccine and Adjuvant Research (CVAR), National Institutes of Biomedical Innovation, Health and Nutrition (NIBIOHN), 7-6-8 Saito-Asagi, Ibaraki-shi, Osaka, 567-0085 Japan

**Keywords:** Biotechnology, Animal biotechnology, Assay systems, Molecular engineering

## Abstract

Chimeric proteins have been widely used to evaluate intracellular protein–protein interactions (PPIs) in living cells with various readouts. By combining an interleukin-3-dependent murine cells and chimeric proteins containing a receptor tyrosine kinase c-kit, we previously established a c-kit-based PPI screening (KIPPIS) system to evaluate and select protein binders. In the KIPPIS components, proteins of interest are connected with a chemically inducible helper module and the intracellular domain of the growth-signaling receptor c-kit, which detects PPIs based on cell proliferation as a readout. In this system, proteins of interest can be incorporated into chimeric proteins without any scaffold proteins, which would be advantageous for evaluating interaction between small peptides/domains. To prove this superiority, we apply KIPPIS to 6 peptide aptamer–polypeptide pairs, which are derived from endogenous, synthetic, and viral proteins. Consequently, all of the 6 peptide aptamer–polypeptide interactions are successfully detected by cell proliferation. The detection sensitivity can be modulated in a helper ligand-dependent manner. The assay results of KIPPIS correlate with the activation levels of Src, which is located downstream of c-kit-mediated signal transduction. Control experiments reveal that KIPPIS clearly discriminates interacting aptamers from non-interacting ones. Thus, KIPPIS proves to be a versatile platform for evaluating the binding properties of peptide aptamers.

## Introduction

Protein–protein interactions (PPIs) are key molecular events that regulate cellular physiology in living cells. Druggability of intracellular targets is highly rewarded but one of the challenges for emerging technologies is to generate novel modalities^1,2^. The PPI modulators should be precisely designed with high affinity and specific target-binding ability to avoid fatal and unpredictable adverse events.

There have been various modalities for inhibiting intracellular aberrant PPIs^3^. Peptide aptamers, which generally have a length of 8–20 residues, are capable of binding to a wider and flatter interface than small molecule inhibitors^4^. Moreover, peptide aptamers have relatively simple structures and can stably bind to intracellular targets compared with antibodies or their fragments whose structural stability depends on forming accurate disulfide bonds^5–7^. Furthermore, peptides can be chemically synthesized^8^ and highly developable by cyclization^9^, stapling^10^, and non-natural amino acids blending^11,12^ to improve half-life in blood by conferring resistance to degradation.

Chimeric proteins have been widely used in protein-fragment complementation assays (PCAs). NanoBiT is a typical example and a powerful tool to evaluate inhibitor candidates against a single PPI pair based on luminescence quenching^13,14^. While the existing PCA systems effectively isolate potential inhibitor candidates via screening small-molecule libraries, they lack enriching superior binders from genetically encoded peptide or antibody libraries. Therefore, there is a need for developing new methods that can spontaneously concentrate superior binders and efficiently eliminate inferior and non-specific ones.

To this end, we previously developed a cell proliferation-based PPI-screening system, represented as c-kit-based PPI screening (KIPPIS)^15^. Basically, the intracellular domain (ICD) of a receptor tyrosine kinase c-kit is fused to the C-terminus of proteins of interest, resulting in cell proliferation induced by the c-kit ICD homodimerization in a PPI-dependent manner. By transducing the chimeric protein genes and hacking signaling pathways in interleukin-3 (IL-3)-dependent Ba/F3 cells, the transductants can proliferate in a PPI-dependent manner even under an IL-3-depleted condition. Through a proof-of-concept experiment of the KIPPIS system, we found that chimeric proteins which contain c-kit ICD play an active role in cell proliferation instead of their IL-3 receptor-mediated proliferative process. In the following research, we additionally fused a ‘helper module’ to chimeric proteins to increase the sensitivity, and completed a basic design of KIPPIS, showing the successful detection of p53–MDM2 interaction^16^. In the present study, we newly test 5 additional PPI pairs in addition to the p53–MDM2 pair to assess the versatility and feasibility of the KIPPIS platform for evaluating intracellularly functional peptide aptamers.

## Results

### The basic design of KIPPIS chimeric proteins

**Figure 1:**
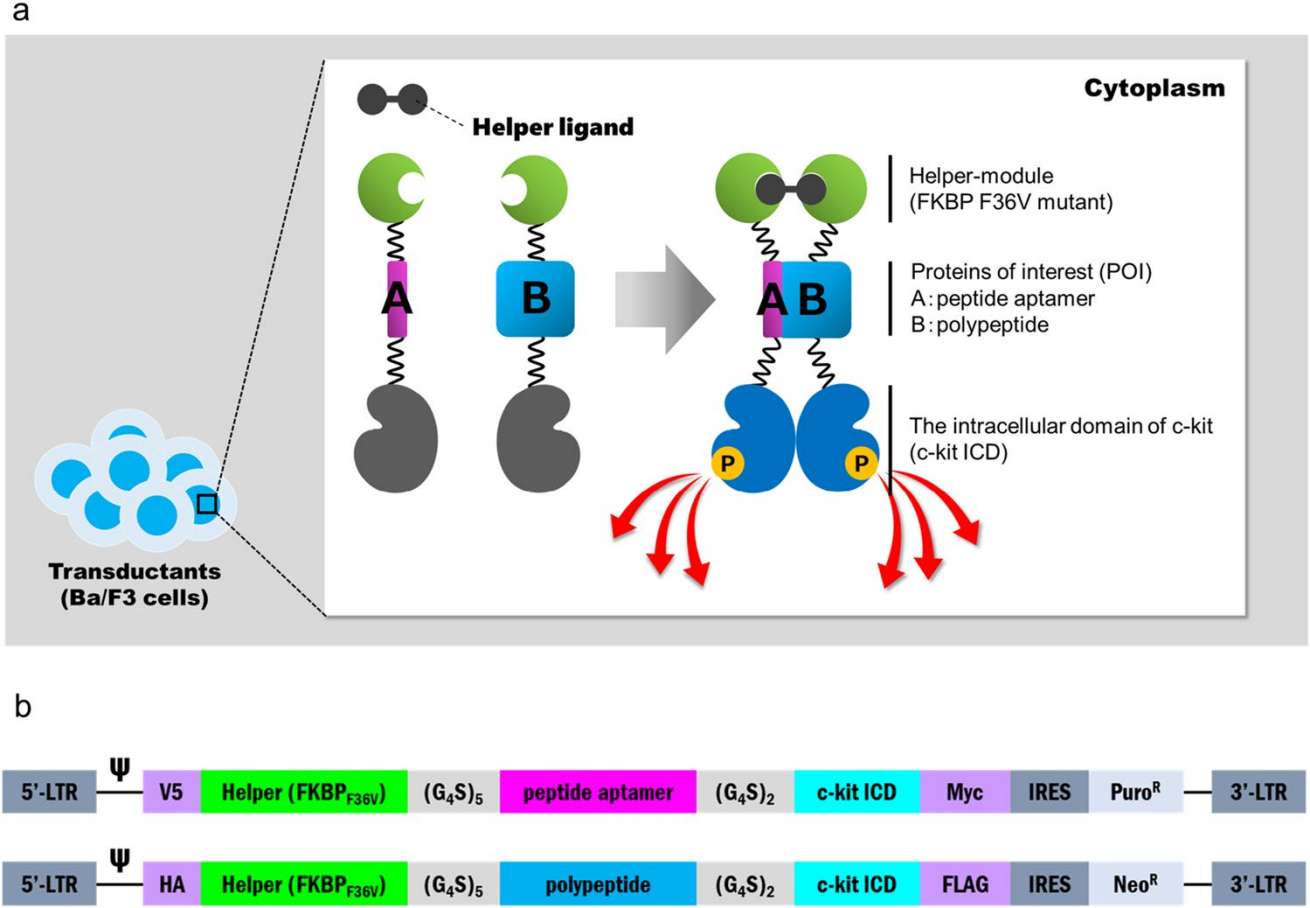
The design and activation concept of KIPPIS for detecting peptide aptamer–polypeptide interaction. (**a**) The constructs of chimeras with a helper module (FKBP_F36V_), a protein of interest [POI: peptide aptamer (A) or polypeptide (B)], and the c-kit intracellular domain (c-kit ICD). The helper ligand supports the formation of stable complexes. Flexible linkers (G_4_S)_5_ and (G_4_S)_2_ were employed to link the helper module/POI and POI/c-kit ICD, respectively. (**b**) The architectures of retroviral vectors. *LTR* long terminal repeat. Psi (Ψ); packaging signal. V5/Myc and HA/FLAG; tag peptides. *IRES* internal ribosome entry site. Puro^R^/Neo^R^; drug resistance gene against puromycin/neomycin.

Proteins of interest (POIs) are flanked by linkers and connected with a helper module (a mutant of FK506-binding protein 12 (FKBP): FKBP_F36V_) at the N-terminus and with c-kit ICD at the C-terminus (Fig. 1; all amino acids sequences summarized in Supplementary Fig. S1). A helper ligand AP20187 conditionally induces homodimerization of the helper modules. Basically, in the KIPPIS system, peptide aptamers do not require any scaffold proteins like thioredoxin^17^. The 6 peptide aptamer–polypeptide pairs tested in this study (detailed information and four out of six structural data are summarized in Supplementary Fig. S2) are grouped into endogenous proteins (p53–MDM2^18^ and EZH2–EED^19,20^), artificial peptide aptamers (PA7–Id1^21,22^ and DIEDML–KIX^23^), and viral peptides (PB1_N_–PA^24,25^ and PB2–PB1_C_^26,27^). The in vitro affinity has been experimentally tested for 3 of 6 peptide aptamer–polypeptide interactions. The p53–MDM2 interaction was evaluated by surface plasmon resonance (SPR) measurements, in which the p53 peptide aptamer is immobilized on sensor chip and the serially diluted MDM2 flowed on the chip (140 ± 5 nM)^18^. To validate the interaction between EZH2 and EED, their binding affinity was measured by isothermal titration calorimetry (ITC) (380 nM)^20^. For determination of the in vitro affinity of the PB1_N_ peptide aptamer (residues 1–15), competitive ELISA using the increasing concentrations against a longer PB1_N_ peptide (residues 1–25)–PA complex (43.3 ± 5.3 nM)^25^. The in vitro affinity values previously reported are summarized (Supplementary Fig. S3a). To establish a constitutively expressing cell line, all genes encoding chimeric proteins were integrated into IL-3-dependent mouse Ba/F3 cells via retroviral vectors followed by drug resistance selection. Transgenes encoding chimeric proteins were introduced pairwise so that the translated products could interact with each other. As a control for the co-transductants with the interacting POI pairs, single transductants and co-transductants with non-interacting POI pairs were prepared for all constructs, in which peptides were shuffled in the groups (Supplementary Fig. S3a).

### Functional evaluation of peptide aptamer- and polypeptide-fused chimeras

**Figure 2:**
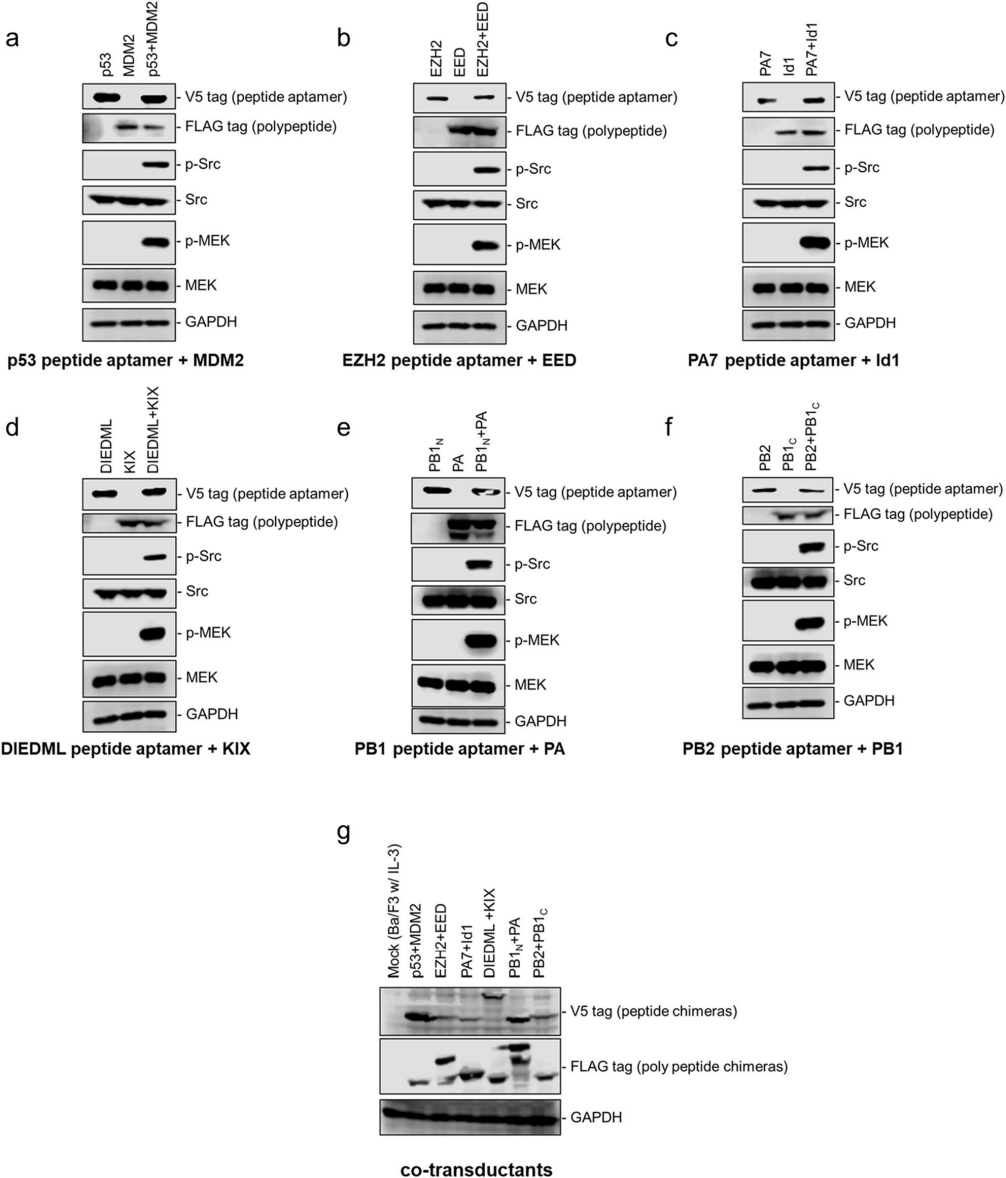
Checking on the expression levels and c-kit ICD-derived signals in the cells expressing the peptide aptamer- and polypeptide-fused chimeras. Cells were depleted and stimulated by 100 nM helper ligand at a fully boosted condition, and the cell lysates were analyzed by western blotting. The chimeras expressed in the Ba/F3 transductants are shown as the abbreviated names. The expression levels of the chimeras were checked by the V5 and FLAG tags for the peptide aptamer- and polypeptide-fused chimeras, respectively. The expression levels and the phosphorylation of endogenous signaling molecules were checked with the following antibodies: anti-pSrc, anti-Src, anti-pMEK, anti-MEK, and anti-GAPDH as a loading control. (**a–f**) Comparison of single and co-transductants for each peptide aptamer/polypeptide pair. (**g**) Co-transductants with different POI pairs were placed side by side. The expression levels of the chimeras and endogenous protein GAPDH were compared in parallel. The images were created by an Image Studio software (ver 4.0; https://www.licor.com/bio/image-studio/) associated with a C-DiGit scanner. Full-length blots are presented in Supplementary Fig. S5.

Western blotting was performed to confirm the expression and activation levels of the chimeric proteins with a full-boosted helper module (Fig. 2a–g). Peptide-fused chimeras (marked with a V5 peptide tag) have equivalent molecular mass values (except DIEDML) and expression levels. Although polypeptide-fused chimeras (marked with a V5 peptide tag) have diverse molecular mass values, no significant difference in the expression level was observed even when the maximum POI (120 kDa; the PA chimera) and the minimum one (76 kDa; the PB1_C_ chimera) were compared.

The activation level of c-kit ICD was assessed by phosphorylation of typical growth- associated signaling molecules: Src family kinases and mitogen-activated protein kinase kinase (MEK)^28,29^. Consequently, all of the co-transductants with the interacting POI pairs clearly induced phosphorylation of both Src and MEK (Fig. 2a–f; p-Src and p-MEK). Since no phosphorylation was detected in single transductants (Fig. 2a–f) and shuffled controls (Supplementary Fig. S3b), the observed phosphorylation was due to neither co-expression nor interaction between helper modules, but PPI-dependent signaling.

### Detection of peptide aptamer–polypeptide interactions based on cell proliferation

**Figure 3:**
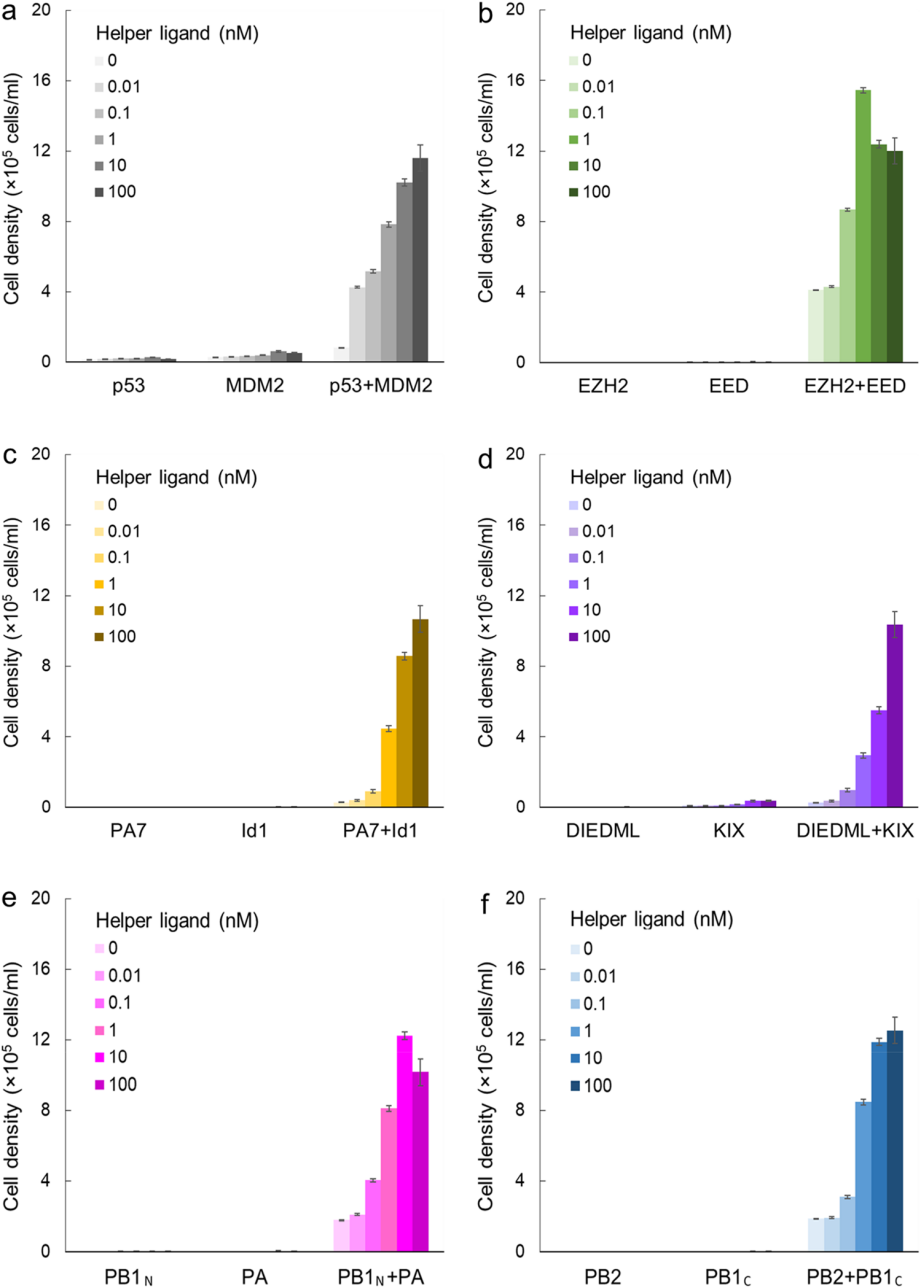
Detection of the endogenous/synthetic/viral peptide aptamer–polypeptide interactions based on cell proliferation. The chimeras expressed in the Ba/F3 transductants are shown as the abbreviated names. A cell proliferation assay was performed for detecting p53–MDM2 (**a**), EZH2–EED (**b**), PA7–Id1 (**c**), DIEDML–KIX (**d**), PB1_N_–PA (**e**), and PB2–PB1_C_ (**f**) interactions with/without the serially diluted helper ligand (0.01, 0.1, 1, 10, 100 nM). The initial cell density was 1 × 10^5^ cells/ml. The viable cell densities after 72 h are indicated as mean ± SD (n = 3, biological replicates).

To test whether the cell growth depends on the helper ligand and PPIs, a cell proliferation assay was performed (Fig. 3). The co-transductants expressing interacting POI pairs but no single transductants proliferated, which was consistent with the phosphorylation patterns in western blotting. Notably, the proliferation levels increased depending on the concentration of the helper ligand, indicating that the helper ligand strongly boosted the sensitivity of KIPPIS. This helper ligand-dependent proliferation was not observed in single transductants, indicating that not only the helper module but also the interaction between POIs was required for the growth signaling. Moreover, cell proliferation was not induced in co-transductants in which the polypeptide chimeras were enforced to pair with the shuffled non-interacting peptide aptamers (Supplementary Fig. S3c). Since our previous study had already shown the p53–MDM2 interaction-dependent cell growth^16^, here we additionally succeeded in inducing cell growth for 5 additional POI pairs.

We also investigated whether Nutlin-3, a validated small-molecule inhibitor against the p53–MDM2 interaction^30^, could be evaluated in KIPPIS as a model. A growth suppression assay was performed together with a negative control cell line, which co-expresses chimeras with exogenous proteins-derived POI pairs (the PB1_N_- and PA-fused chimeras). Consequently, cell growth was clearly inhibited by addition of Nutlin-3 in the cells co-expressing the p53- and MDM2-fused chimeras (Supplementary Fig. S4). On the other hand, the negative control cells expressing the PB1_N_- and PA-fused chimeras maintain relative viable cell density at high levels (more than 100% compared to that without Nutlin-3) throughout all Nutlin-3 concentrations tested. The half maximal inhibitory concentration (IC_50_) is defined as the concentration at which a 50% reduction in relative viable cell density occurred in KIPPIS. The IC_50_ value of KIPPIS (3.3 ± 0.2 µM) was consistent with the sensitivity to Nutlin-3 (IC_50_ = 2.21 to 2.47 μM) which was previously reported based on a growth suppression assay using gastric cancer cell lines^31^. Thus, KIPPIS could reproduce the p53–MDM2 interaction at the same level as endogenous proteins, and might be compatible with other in vitro assays evaluating endogenous intracellular PPIs.

## Discussion

KIPPIS successfully detected interaction of endogenous, synthetic, and viral aptamers against their binding targets with various molecular sizes. Thus, 6 aptamer–polypeptide pairs in total were proliferatively detectable in a helper ligand concentration- and PPI-dependent manner. Surprisingly, the helper module boosted cell proliferation in all peptide aptamer–polypeptide interactions. Even with 100 nM helper ligand, off-target proliferation was below the initial cell concentration and the ratio to on-target proliferation was less than 5% in the single transductants and shuffled controls.

KIPPIS has a history of improving sensitivity by fusing the helper module, and its sensitivity can be adjusted by the concentration of the helper ligand AP20187. Even without the helper ligand, KIPPIS was able to detect EZH2 peptide aptamer–EED polypeptide interaction (*K*_D_ = 380 nM) and other two interacting pairs (PB1_N_–PA and PB2–PB1_C_). The interactions of other three interacting pairs were detectable at least by adding 0.1 nM of the helper ligand. Therefore, weaker binding (e.g. μM order of *K*_D_) could be detected by adding the helper ligand at a maximal concentration (e.g. 100 nM).

KIPPIS is featured by chemically tunable sensitivity, low background, and wide allowable range of target proteins. We previously optimized the length of flexible linkers between the helper module and POI and between POIs and c-kit ICD^16^. In this study, our findings indicate that the chimeric proteins have an extensive capacity for accommodating various targets. Thus, the flexible linkers between the domains of the chimeric proteins would play a pivotal role in the high capacity as well as low background signals. As a concern in future applications, the length of linkers may be re-examined when we use full length proteins with a large molecular mass more than 60 kDa. In the case of synthetic library screening, the linkers should be optimized depending on POIs because the expression level bias among the clones would affect the outcome of the screening in a long-term cell culture.

In this study, we evaluated the versatility of KIPPIS using existing peptides instead of library screening. Though KIPPIS is an advantageous tool as a selector, the bottleneck may be a scalability issue because the KIPPIS screening in this study was performed in a small-scale batch culture with manual operations. Therefore, the robustness is required to handle a synthetic library with 10 amino acids or more mutations. Regarding this point, KIPPIS may be applied for large-scale screening by combining next-generation technologies: in silico prediction to narrow down library size^32^ or a virus-based continuous evolution system in mammalian cells to actuate DNA sequences, which the authors dub “VEGAS”^33,34^.

## Materials and methods

### Plasmid construction

All plasmids constructed in this study are retroviral plasmids with a pMK backbone. All oligonucleotides used for constructing the plasmids and the resultant plasmids are listed in Supplementary Tables S1 and S2.

Among 5 peptides other than the template p53, each plasmid encoding either EZH2 or PB2 peptide was constructed by inserting the corresponding DNA sequence at a predetermined POI position using 5 × In-Fusion HD Enzyme Premix (Takara Bio, Shiga, Japan). A plasmid encoding PB1_N_ was constructed by mutagenesis using PrimeSTAR Max DNA polymerase (Takara Bio). For convenience of PCR, a DNA fragment encoding a (G_4_S)_2_ linker was once deleted from the plasmid, and then (G_4_S)_2_ was inserted again downstream of the target peptide by PrimeSTAR mutagenesis. A DNA fragment created by hybridizing two oligonucleotides encoding a PA7 peptide or by cleaving a plasmid encoding DIEDML with MluI-HF and MfeI-HF (New England Biolabs, Ipswich, MA) was ligated with a host plasmid (EZH2) cleaved with the same restriction enzymes using Ligation High Ver.2 (Toyobo, Osaka, Japan).

Among 5 polypeptides other than the template MDM2, PCR fragments encoding EED, PA, and PB1_C_ were prepared using deposited plasmids or synthetic genes described below as templates. Purified PCR fragments were fused at a predetermined POI position as described above for constructing the plasmids encoding EZH2 and PB2. As well as PB1_N_, a DNA fragment encoding a (G_4_S)_2_ linker was once deleted and inserted again by PrimeSTAR mutagenesis. Id1 and KIX were prepared by restriction enzyme cleavage and subsequent ligation as described above for constructing the plasmids encoding PA7 and DIEDML.

Synthetic genes encoding EED (residues 81–441), PA (residues 257–716), and PB1_C_ (residues 678–757) cloned in pUC57 were purchased from Genscript (Piscataway, NJ). The plasmid encoding Id1 (#16061 pcDNA3 hId1) was purchased from Addgene (Watertown, MA). The plasmids encoding DIEDML and KIX were kindly provided by Dr. H. Bito (Department of Neurochemistry, The University of Tokyo). NEB Turbo *E. coli* competent cells (New England Biolabs) were used for cloning the plasmids. An antibiotic ampicillin was purchased from Thermo Fisher Scientific (Waltham, MA). The basic experimental procedures for transformation of *E. coli* and extraction of plasmid DNA were performed according to a standard protocol.

### Cell lines, transfection, and transduction

The mouse-derived interleukin-3 (IL-3)-dependent cell line Ba/F3 (RCB0805) was purchased from RIKEN Cell Bank (Ibaraki, Japan). Ba/F3 was cultured in RPMI-1640 (RPMI) medium (Nissui Pharmaceutical, Tokyo, Japan) containing 10% v/v fetal bovine serum (FBS; Biowest, Paris, France) and 1 ng/ml mouse IL-3 (R&D Systems, Minneapolis, MN) at 37 °C in a 5% CO_2_ atmosphere. Plat-E, a retrovirus packaging cell, was used to produce retroviral vectors for transduction into Ba/F3 cells. Plat-E was cultured in Dulbecco modified Eagle's medium (DMEM; Nissui Pharmaceutical) containing 10% v/v FBS, 1 µg/ml puromycin (Sigma-Aldrich, St. Louis, MO) and 10 µg/ml blasticidin (Kaken Pharmaceutical, Tokyo, Japan) at 37 °C in a 10% CO_2_ atmosphere.

Plat-E cells were seeded on a 6-well plate at 5.0 × 10^5^ cells/ml. Lipofectamine LTX (Thermo Fisher Scientific) was used for lipofecting Plat-E cells with ethanol-precipitated purified plasmid. Three micrograms of plasmid DNA was resuspended with 150 µl of Opti-MEM I Reduced Serum Medium (Thermo Fisher Scientific) with 3 µl of Plus Reagent (Thermo Fisher Scientific). The same volume of Opti-MEM was mixed with 7.5 µl of Lipofectamine LTX, which was subsequently mixed gently with the mixture of the Plus Reagent and plasmid DNA. The resultant mixture was added dropwise to Plat-E cells cultured in each well of a 6-well plate. The lipofected cells were cultured at overnight. The day-2 culture supernatant was used for retrovirally transducing Ba/F3 cells in a 24-well plate pre-coated with 25 µg/ml RetroNectin (Takara Bio).

The retroviral plasmids encode the gene of a chimeric protein of interest, followed by an internal ribosomal entry site (IRES) and a puromycin or neomycin resistance gene (Puro^R^ or Neo^R^). Hence, a stable expression strain harboring the chimeric protein was established through antibiotics selection using 2 µg/ml puromycin and/or 800 µg/ml G418 (FUJIFILM Wako Pure Chemical, Osaka, Japan).

### Western blot analysis

Subcultured cells (5.0 × 10^6^) were washed 3 times with PBS, resuspended in 10 ml RPMI medium without IL-3 and antibiotics, and cultured in a 10 cm dish for 12 h (depletion). The depleted cells (1.0 × 10^6^) were collected in microtubes and washed with PBS once, resuspended in 1 ml RPMI medium with or without 100 nM AP20187, and cultured at 37 °C for 30 min (stimulation). The stimulated cells were washed twice with 2 mM Na_3_VO_4_/ice-cold PBS, and incubated on ice for 10 min after lysing with 100 μl of lysis buffer (20 mM HEPES (pH 7.5), 150 mM NaCl, 10% glycerol, 1% Triton X-100, 1.5 mM MgCl_2_, 1 mM EGTA, 1 mM Na_3_VO_4_, 10 μg/ml aprotinin, 10 μg/ml leupeptin). The cell lysate was centrifuged at 21,500*g* at 4 °C for 10 min, and the supernatant was collected to another microtube and mixed with 33 μl of 4 × Laemmli’s sample buffer and heated up to 98 °C for 5 min using Dry Thermo Unit DTU-28 (TAITEC, Saitama, Japan).

SDS-PAGE (8 or 12% acrylamide gel) was performed to separate proteins comprising the lysates. The proteins separated on the gel were transferred onto a nitrocellulose membrane (GE Healthcare, Chicago, IL) in wet conditions. The membrane was blocked either with 5% skim milk (FUJIFILM Wako Pure Chemical) for detection of V5 and FLAG tags or with 3% bovine serum albumin (Sigma-Aldrich) for detection of phospho-Src, whole Src, phospho-MEK, whole MEK, and glyceraldehyde 3-phosphate dehydrogenase (GAPDH). The blots were specifically probed with rabbit primary antibodies (Supplementary Table S3). A horseradish peroxidase (HRP)-conjugated goat anti-rabbit IgG (Thermo Fisher Scientific) was used as a secondary antibody. Luminata Forte Western HRP Substrates (Merck Millipore, Burlington, MA) and a C-DiGit scanner (LI-COR Biosciences, Lincoln, NE) were used for luminescence generation and detection. The images were created by an Image Studio software (ver 4.0; https://www.licor.com/bio/image-studio/) associated with a C-DiGit scanner. All blots were rendered as single images with a default setting in the software, where the image display settings do not alter raw data and signal intensities.

### Proliferation assay

Subcultured cells were washed with PBS twice to remove IL-3 in the culture medium. Cells were seeded into 24-well plates at 1 × 10^5^ cells/ml with or without serial concentrations of AP20187 or Nutlin-3 (FUJIFILM Wako Pure Chemical) for 72 h. Flow cytometry was employed for cell counting. Before measurement, the sample mixtures (100 µl of cell suspension mixed with 4 µl of Flow-Count beads (Beckman Coulter, Brea, CA) and 46 µl of PBS containing 1 µg/ml of propidium iodide (Sigma‐Aldrich) at a final concentration) were transferred to 96-well plates and the number of cells was counted with a FACSCalibur flow cytometer (Becton–Dickinson, Franklin Lakes, NJ) calibrated by the count of Flow-Count beads. The data was processed by the software FlowJo v7.6.5 (Becton–Dickinson). The EC_50_ values of the helper ligand was calculated based on the results of the proliferation assay by curve fitting to the logistic function (Rodbard) using the software ImageJ v1.53 (National Institutes of Health, Bethesda, MD). The EC_50_ values were listed with the previously reported in vitro affinity values (Supplementary Fig. S3a).

### Molecular modeling

Protein illustrations based on crystal structures (Supplementary Fig. S2) were drawn using a molecular drawing software PyMOL (Schrödinger, New York, NY).

## Supplementary Information

Supplementary Information.
