# Supplementary material for: Evolution of KIPPIS as a versatile platform for evaluating intracellularly functional peptide aptamers
Source: Sci Rep. 2021 Jun 3;11:11758. doi: 10.1038/s41598-021-91287-z (PMC8175380; doi:10.1038/s41598-021-91287-z)
Supplement: Supplementary file 1 — Supplementary Information. [file 41598_2021_91287_MOESM1_ESM.pdf]

Supplementary Information for

Evolution of KIPPIS as a versatile platform for evaluating intracellularly functional peptide aptamers

Daiki Kashima[1], Masahiro Kawahara[1, 2, *]

Affiliations:

[1]Department of Chemistry and Biotechnology, Graduate School of Engineering, The University of Tokyo, 7-3-1 Hongo, Bunkyo-ku, Tokyo 113-8656, Japan.

[2]Laboratory of Cell Vaccine, Center for Vaccine and Adjuvant Research (CVAR), National Institutes of Biomedical Innovation, Health and Nutrition (NIBIOHN), 7-6-8 Saito-Asagi, Ibaraki-shi, Osaka 567-0085, Japan.

*Correspondence to: m-kawahara@nibiohn.go.jp

This PDF file includes:

Tables S1 to S3

Figs. S1 to S5

Table S1. Oligonucleotides used for plasmid construction

Name	Sequence (5' - 3')
EZH2-HOST_Fw	ccagtcacgcgtTCGACGACCTACAAATATTTACAGAAACCCATGTATGAAGTACAGTGG
EZH2-HOST_Rv	ggtcttcaattgTGAGCCACCTCCTCCGCTGC
EZH2-insert_Fw	ggtggctcacaattgAAGACCATGTTTTCCAGCAACCGCC
EZH2-insert_Rv	ggtcgtcgaacgcgtGACTGGTTGAATCCTCCGCTGTTTCC
EZH2-addlinker_Fw	ggcggttcaggcggtggcgggTCGACGACCTACAAATATTTACAGAAACCCATGTATG
EZH2-addlinker_Rv	accgcctgaaccgcctccaccACGCGTGACTGGTTGAATCCTC
PB2n_HOST_Fw	ggcggctcgagcTCGACGACCTACAAATATTTACAGAAACCCA
PB2n_HOST_Rv	catacgcgtTGAGCCACCTCCTCCGCTG
PB2n_insert_Fw	gtggctcaACGCGTATGGAGCGGATC
PB2n_insert_Rv	cgagctcgagccGCCAGAGGTGTACTTCTTGATGATG
add_G4S2_PB2n_Fw	ggcggttcaggcggtggcgggTCGACGACCTACAAATATTTACAGAAACCC
add_G4S2_PB2n_Rv	accgcctgaaccgcctccaccGCTCGAGCCGCCAGAGGT
H5B-ver2_Fw	caccctgctgttcctgaaggtgcctgcccagTCGACGACCTACAAATATTTACAGAAACC
H5B-ver2_Rv	caggaacagcagggtggggttcacgtccatTGAGCCACCTCCTCCGCT
H5B-G4S2_Fw	GTGGAGGCggttcaggcggtggcgggTCGACGACCTACAAATATTTACAGAAA
H5B-G4S2_Rv	CTGAACCgcctccaccgctcgagcccCTGGGCAGGCACCTTCAG
PA7_Fw	aattgCTGAGCGCCATGGCCGCCACCCTGTTCGCCGAGCTGGGCTGCCACCTGAGCAGGTGGATGa
PA7_Rv	cgcgtCATCCACCTGCTCAGGTGGCAGCCCAGCTCGGCGAACAGGGTGGCGGCCATGGCGCTCAGc
EED-HOST_Fw	ctgcggacgcgtTCGACGACCTACAAATATTTACAGAAACCCATGTATGAAGTACAGTGG
EED-HOST_Rv	gaatgacaattgTGAGCCACCTCCTCCGCTGC
EED-insert_Fw	ggtggctcacaattgTCATTCAAGTGCGTCAATAGCCTCAAGGAAGACCACAAC
EED-insert_Rv	ggtcgtcgaacgcgtCCGCAGCCTATCCCACCGCC
EED-addlinker_Fw	ggcggttcaggcggtggcgggTCGACGACCTACAAATATTTACAGAAACCCATGTATG
EED-addlinker_Rv	accgcctgaaccgcctccaccACGCGTCCGCAGCCTATCCC
H5A-HOST-ver2_Fw	ccctgtccTCGACGACCTACAAATATTTACAGAAACCC
H5A-HOST-ver2_Rv	ccgttcatTGAGCCACCTCCTCCGCT
H5A-insert-ver2_Fw	ccctgtccGGCTCGAGCGGTGGAGGCG
H5A-insert-ver2_Rv	gtcgtcgaGGACAGGGCGTGTGTCA
H5A-G4S2_Fw	GTGGAGGCggttcaggcggtggcgggTCGACGACCTACAAATATTTACAGAAA
H5A-G4S2_Rv	CTGAACCgcctccaccgctcgagccGGACAGGGCGTGTGTCAG
PB1c_HOST_Fw	gaaaggctcgagcTCGACGACCTACAAATATTTACAGAAACCCA
PB1c_HOST_Rv	ctacgcgtTGAGCCACCTCCTCCGCTG
PB1c_insert_Fw	gtggctcaACGCGTAGTCAGAGAGGGGTC
PB1c_insert_Rv	cgtcgagctcgagccTTTCTGGCGTCGCAGTTCT
add_G4S2_PB1c_Fw	ggcggttcaggcggtggcgggTCGACGACCTACAAATATTTACAGAAACCC
add_G4S2_PB1c_Rv	accgcctgaaccgcctccaccGCTCGAGCCTTTCTGGCG
PA7_bait_Fw	gggcaattgAAAGTCGCCAGTGGCAGCACC
PA7_bait_Rv	gggacgcgtGCGACACAAGATGCGATCGTCCGC

Table S2. Plasmids used for retrovirus production

Name	Detailed construction (5' - 3')
p53*	pMK-FKBP$_{F36V}$-(G$_4$S)$_5$-p53 $_{15\text{-}29}$-(G$_4$S)$_2$-c-kit ICD-IRES-Puro^R
MDM2*	pMK-FKBP$_{F36V}$-(G$_4$S)$_5$-MDM2 $_{17\text{-}125}$-(G$_4$S)$_2$-c-kit ICD-IRES-Neo^R
EZH2	pMK-FKBP$_{F36V}$-(G$_4$S)$_5$-EZH2 $_{40\text{-}68}$-(G$_4$S)$_2$-c-kit ICD-IRES-Puro^R
EED	pMK-FKBP$_{F36V}$-(G$_4$S)$_5$-EED $_{81\text{-}441}$-(G$_4$S)$_2$-c-kit ICD-IRES-Neo^R
PA7	pMK-FKBP$_{F36V}$-(G$_4$S)$_5$-PA7-(G$_4$S)$_2$-c-kit ICD-IRES-Puro^R
Id1	pMK-FKBP$_{F36V}$-(G$_4$S)$_5$-Id1 $_{1\text{-}155\ (full)}$-(G$_4$S)$_2$-c-kit ICD-IRES-Neo^R
DIEDML	pMK-FKBP$_{F36V}$-(G$_4$S)$_5$-DIEDML $_{1\text{-}286}$-(G$_4$S)$_2$-c-kit ICD-IRES-Puro^R
KIX	pMK-FKBP$_{F36V}$-(G$_4$S)$_5$-KIX $_{553\text{-}679}$-(G$_4$S)$_2$-c-kit ICD-IRES-Neo^R
PB1$_N$	pMK-FKBP$_{F36V}$-(G$_4$S)$_5$-PB1 $_{1\text{-}15}$-(G$_4$S)$_2$-c-kit ICD-IRES-Puro^R
PA	pMK-FKBP$_{F36V}$-(G$_4$S)$_5$-PA $_{257\text{-}716}$-(G$_4$S)$_2$-c-kit ICD-IRES-Neo^R
PB2	pMK-FKBP$_{F36V}$-(G$_4$S)$_5$-PB2 $_{1\text{-}37}$-(G$_4$S)$_2$-c-kit ICD-IRES-Puro^R
PB1$_C$	pMK-FKBP$_{F36V}$-(G$_4$S)$_5$-PB1 $_{678\text{-}757}$-(G$_4$S)$_2$-c-kit ICD-IRES-Neo^R

* p53 and MDM2 are previously described and used as a template in this study.

Table S3. Primary antibodies used for western blot analysis

Name	Manufacturer
Anti-V5 Epitope tag (Rabbit Polyclonal)	Merck Millipore (Burlington, MA)
rabbit anti-DYKDDDDK Tag Polyclonal Antibody	Bethyl Laboratories (Billerica, MA)
Phospho-Src Family (Tyr416) (D49G4) Rabbit mAb	Cell Signaling Technology (Danvers, MA)
Non-phospho-Src (Tyr416) (7G9) Mouse mAb	Cell Signaling Technology
P-MEK1/2 (S217/221) Rabbit mAb	Cell Signaling Technology
MEK 1/2 (D15A) Rabbit mAb	Cell Signaling Technology
GAPDH rabbit polyclonal IgG	Santa Cruz Biotechnology (Dallas, TX)

HA-Helper(FKBP$_{F36V}$)-(G$_4$S)$_5$-peptide aptamer-(G$_4$S)$_2$-c-kit ICD-Myc
(610 aa, 66.7 kDa)

M**GKPIPNPLLGLDST**GSGGVQVETISPGDGRTFPKRGQTCVVHYTGMLEDGKKVDSSRDR
NKPFKFMLGKQEVIRGWEEGVAQMSVGQRAKLTISPDYAYGATGHPGIIPPHATLVFDVELLK
LEGGGGSGGGGSGGGGSGGGGSGGGGSQL **peptide aptamer**
TRGGGGSGGGGSTTYKYLQKPMYEVQWKVVEEINGNNYVYIDPTQLPYDHKWEFPRNRLS
FGKTLGAGAFGKVVEATAYGLIKSDAAMTVAVKMLKPSAHLTEREALMSELKVLSYLGNHMN
IVNLLGACTIGGPTLVITEYCCYGDLLNFLRRKRDSFICSKQEDHAEAALYKNLLHSKESSCSDST
NEYMDMKPGVSYVVPTKADKRRSVRIGSYIERDVTPAIMEDDELALDLEDLLSFSYQVAKGM
AFLASKNCIHRDLAARNILLTHGRITKICDFGLARDIKNDSNYVVKGNARLPVKWMAPESIFN
CVYTFESDVWSYGIFLWELFSLGSSPYPGMPVDSKFYKMIKEGFRMLSPEHAPAEMYDIMKT
CWDADPLKRPTFKQIVQLIEKQISESTNHIYSNLANCSPNRQKPVVDHSVRINSVGSTASSSQ
PLLVHDDVID**EQKLISEEDL**

p53 (15 aa, 1.8 kDa)
SQETFSDLWKLLPEN

EZH2 (30 aa, 3.7 kDa)
KTMFSSNRQKILERTETLNQEWKQRRIQPV

PA7 (20 aa, 2.2 kDa)
LSAMAATLFAELGCHLSRWM

DIEDML (289 aa (6 aa), 30.2 kDa (0.7 kDa))
**LTGMESGAENQQSGDAAVTEAENQQMTVQAQPQIATLAQVSMPAAHATSSAPTVTLVQ
LPNGQTVQVHGVIQAAQPSVIQSPQVQTVQISTIAESEDSQESVDSVTDSQKRREILSDIED
MLKILNDLSSDAPGVPRIEEEKSEEETSAPAITTVTVPTPIYQTSSGQYIAITQGGAIQLANN
GTDGVQGLQTLTMTNAAATQPGTTILQYAQTTDGQQILVPSNQVVVQAASGDVQTYQIR
TAPTSTIAPGVVMASSPALPTQPAEEAARKREVRLMKNREAARECGS**

PB1$_N$ (15 aa, 1.7 kDa)
MDVNPTLLFLKVPAQ

PB2 (37 aa, 4.5 kDa)
TRMERIKELRNLMSQSRTREILTKTTVDHMAIIKKYT

V5-Helper(FKBP$_{F36V}$)-(G$_4$S)$_5$-polypeptide-(G$_4$S)$_2$-c-kit ICD-FLAG
(603 aa, 66.2 kDa)

MYPYDVPDYAGSGGVQVETISPGDGRTFPKRGQTCVVHYTGMLEDGKKVDSSRDRNKPFK
FMLGKQEVIRGWEEGVAQMSVGQRAKLTISPDYAYGATGHPGIIPPHATLVFDVELLKLEGG
GGSGGGGSGGGGSGGGGSGGGGSQL polypeptide
TRGGGGSGGGGSTTYKYLQKPMYEVQWKVVEEINGNNYVYIDPTQLPYDHKWEFPRNRLS
FGKTLGAGAFGKVVEATAYGLIKSDAAMTVAVKMLKPSAHLTEREALMSELKVLSYLGNHMN
IVNLLGACTIGGPTLVITEYCCYGDLLNFLRRKRDSFICSKQEDHAEAALYKNLLHSKESSCSDST
NEYMDMKPGVSYVVPTKADKRRSVRIGSYIERDVTPAIMEDDELALDLEDLLSFSYQVAKGM
AFLASKNCIHRDLAARNILLTHGRITKICDFGLARDIKNDSNYVVKGNARLPVKWMAPESIFN
CVYTFESDVWSYGIFLWELFSLGSSPYPGMPVDSKFYKMIKEGFRMLSPEHAPAEMYDIMKT
CWDADPLKRPTFKQIVQLIEKQISESTNHIYSNLANCSPNRQKPVVDHSVRINSVGSTASSSQ
PLLVHDDVIDDYKDDDDK

MDM2 (96 aa, 11.3 kDa)
ASEQETLVRPKPLLLKLLKSVGAQKDTYTMKEVLFYLGQYIMTKRLYDEKQQHIVYCSNDLL
GDLFGVPSFSVKEHRKIYTMIYRNLVVVNQQ

EED (361 aa, 41.6 kDa)
SFKCVNSLKEDHNQPLFGVQFNWHSKEGDPLVFATVGSNRVTLYECHSQGEIRLLQSYVDA
DADENFYTCAWTYDSNTSHPLLAVAGSRGIIRIINPITMQCIKHYVGHGNAINELKFHPRDP
NLLLSVSKDHALRLWNIQTDTLVAIFGGVEGHRDEVLSADYDLLGEKIMSCGMDHSLKLW
RINSKRMMNAIKESYDYNPNKTNRPFISQKIHFPDFSTRDIHRNYVDCVRWLGDLILSKSC
ENAIVCWKPGKMEDDIDKIKPSESNVTILGRFDYSQCDIWYMRFSMDFWQKMLALGNQ
VGKLYVWDLEVEDPHKAKCTTLTHHKCGAAIRQTSFSRDSSILIAVCDDASIWRWDRLR

Id1 (129 aa, 14.3 kDa)
MKVASGSTATAAAGPSCALKAGKTASGAGEVVRCLSEQSVAISRCAGGAGARLPALLDEQ
QVNVLLYDMNGCYSRLKELVPTLPQNRKVSKVEILQHVIDYIRDLQLELNSESEVGTPGGRG
LPVRAPLSTLNGEISALTAEAACVPADDRILCR

KIX (479 aa, 54.8 kDa)
GLTGLGATNPLMNDGSNSGNIGSLSTIPTAAPPSSTGVRKGWHEHVTQDLRSHLVHKLVQ
AIFPTPDPAALKDRRMENLVAYAKKVEGDMYESANSRDEYYHLLAEKIYKIQKELEEKRRSR
LHKQGIL

PA (80 aa, 9.5 kDa)
MNGYIEGKLSQMSKEVNARIEPFLKTTPRPLRLPNGPPCSQRSKFLLMDALKLSIEDPSHEG
EGIPLYDAIKCMRTFFGWKEPNVVKPHEKGINPNYLLSWKQVLAELQDIENEEKIPKTKNM
KKTSQLKWALGENMAPEKVDFDDCKDVGDLKQYDSDEPELRSLASWIQNEFNKACELTD
SSWIELDEIGEDVAPIEHIASMRRNYFTSEVSHCRATEYIMKGVYINTALLNASCAAMDDF
QLIPMISKCRTKEGRRKTNLYGFIIKGRSHLRNDTDVVNFVSMEFSLTDPRLEPHKWEKYCV
LEIGDMLIRSAIGQVSRPMFLYVRTNGTSKIKMKWGMEMRRCLLQSLQQIESMIEAESSV
KEKDMTKEFFENKSETWPIGESPKGVEESSIGKVCRTLLAKSVFNSLYASPQLEGFSAESRKL
LLIVQALRDNLEPGTFDLGGLYEAIEECLINDPWVLLNASWFNSFLTHALS

PB1$_C$ (80 aa, 9.5 kDa)
SQRGVLEDEQMYQRCCNLFEKFFPSSSYRRPVGISSMVEAMVSRARIDARIDFESGRIKKE
EFTEIMKICSTIEELRRQK

Fig. S1. Full amino acid sequence of chimeric proteins. Single-letter amino acid notations are colored corresponding to respective domains as shown in the chimeric construct names at the top of the sequence list. The peptide aptamer/polypeptide boxes can be exchanged for the POIs shown below. The molecular mass of the chimeric proteins can be calculated by the sum of the framework chimeric protein (66.7/66.2 kDa; without peptide aptamer/polypeptide) and each POI. QL (Gln-Leu) and TR (Thr-Arg) displayed in gray indicate amino acids (2 aa) encoded by restriction enzyme sites (6 bp).

a

pairs of interacting peptide	residues	polypeptides	residues	PDB ID
p53	40–68	MDM2	17–125	1YCR
EZH2	40–68	EED	81–441	2QXV
PA7	synthetic	Id1	1–155 (full length)	N.A.
DIEDML	synthetic	KIX	1–286	N.A.
$PB1_N$	1–15	PA	257–716	2ZNL
PB2	1–37	$PB1_C$	678–757	2ZTT

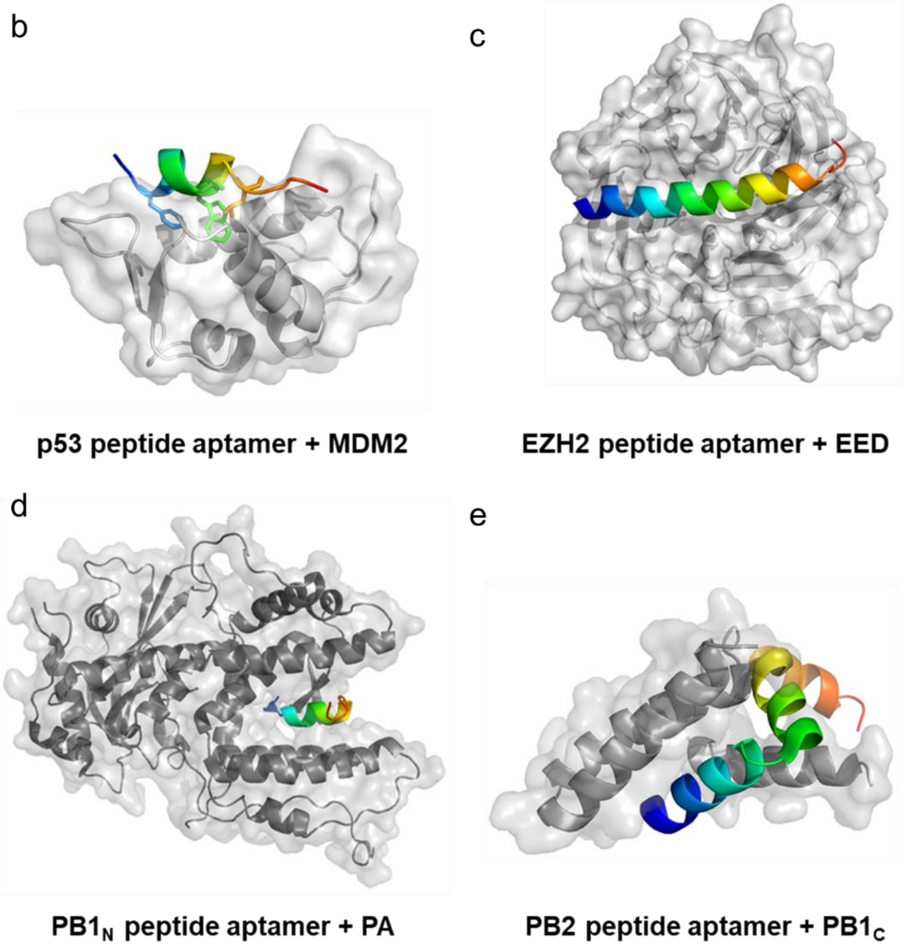

Fig. S2. Information on peptide aptamers and polypeptides. **a,** Amino acid residue numbers and PDB IDs are listed. **b–e,** Crystal structure of the peptide aptamer (colored)‑polypeptide (monochrome) complexes. The structural information of artificial peptide aptamers (PA7 and DIEDML) has not been reported.

a

NC#	polypeptides	shuffled negative controls (**NC**)	pairs of interacting peptide	affinity (nM)		EC_{50} (nM) of helper ligand
1	MDM2	EZH2	p53	140±5	SPR (K_D)	0.21
2	EED	p53	EZH2	380	ITC (K_D)	0.10
3	Id1	DIEDML	PA7		N.A.	1.70
4	KIX	PA7	DIEDML		N.A.	7.82
5	PA	PB2	$PB1_N$	43.3±5.3	ELISA (IC_{50})	0.40
6	$PB1_C$	$PB1_N$	PB2		N.A.	0.63

b

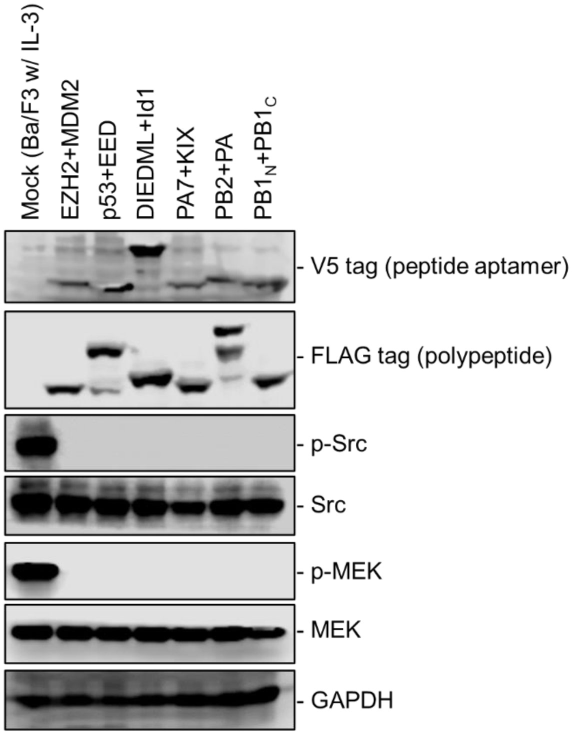

c

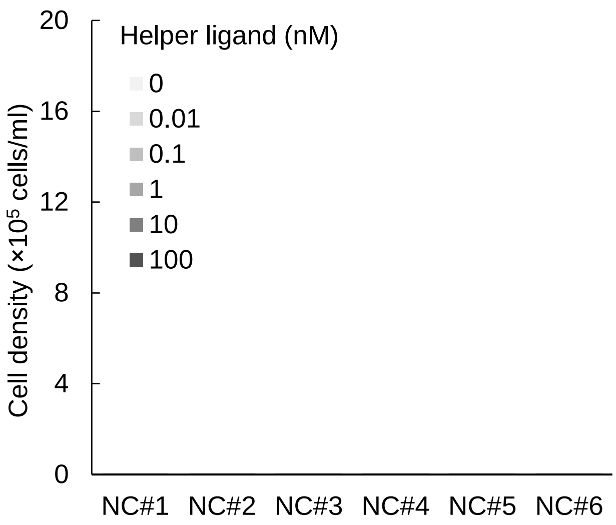

Fig. S3. Configuration of shuffled negative control and experimental results. a, Six peptide aptamers are divided into three groups by endogenous, synthetic, and viral-derived peptides. To assess non-specific binding, peptide aptamers are replaced within the groups (as shown in the list), which are named "shuffled negative controls (NC)". The reported affinity and its measurement method in the interacting pair are listed. **b,** Shuffled negative controls hardly affect the phosphorylation levels of MEK and Src compared with a positive control (parental Ba/F3 cells stimulated with IL-3). As in the case of interacting POI pairs, cells were depleted and stimulated by 100 nM helper ligand at a fully boosted condition, and the cell lysates were analyzed by western blotting. The chimera pairs expressed in the Ba/F3 transductants are shown

as the number of shuffled negative controls (NC#). The expression levels of the chimeras were checked by the V5 and FLAG tags for the peptide aptamer- and polypeptide-fused chimeras, respectively. The expression levels and the phosphorylation of endogenous signaling molecules were checked with the following antibodies: anti-pSrc, anti-Src, anti-pMEK, anti-MEK, and GAPDH as a loading control. The images were created by an Image Studio software (ver 4.0; https://www.licor.com/bio/image-studio/) associated with a C-DiGit scanner. Full-length blots are presented in Supplementary Information, Fig. S5. **c,** Shuffled negative controls hardly induced cell proliferation. The chimera pairs expressed in the Ba/F3 transductants are shown as the number of shuffled negative controls (NC#). A cell proliferation assay was performed for detecting with/without serially diluted helper ligand (0.01, 0.1, 1, 10, 100 nM). The initial cell density was 1×10^5 cells/ml. The viable cell densities after 72 h are indicated as mean ± SD (n=3, biological replicates).

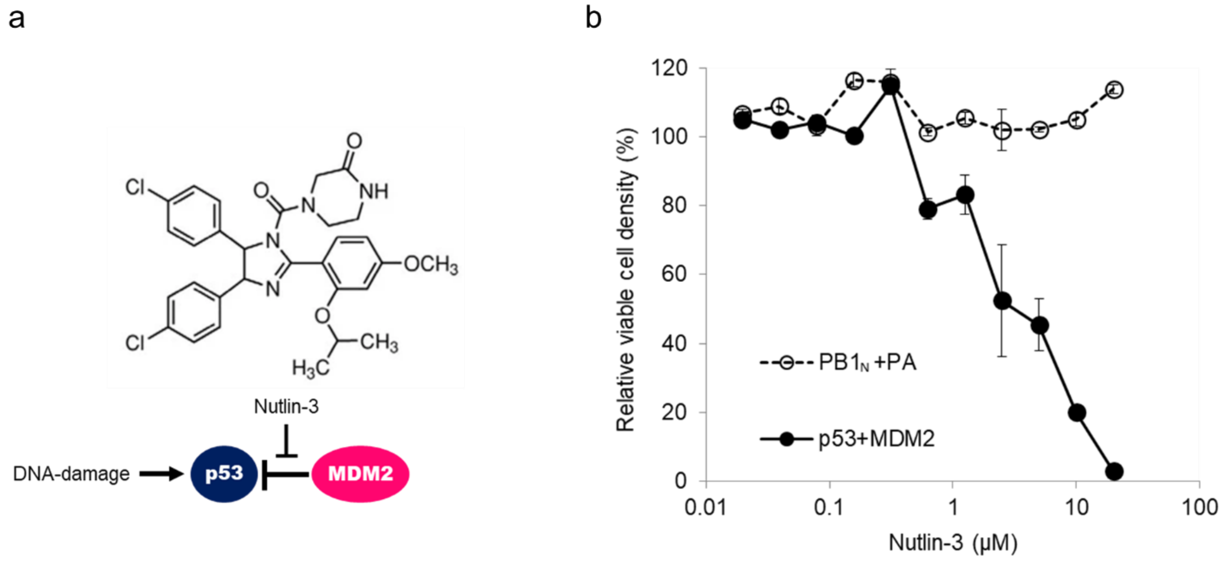

Fig. S4. Mechanism of action of Nutlin-3 and a result of growth suppression assay. a, Nutlin-3 exerts an anticancer effect by improving the physiological function of p53.The p53 protein acts as a transcription factor, whose physiological functions are known to activate damaged DNA repair proteins, control the cell cycle, and induce apoptosis when DNA is irreparably damaged. On the other hand, MDM2 works as a suppressor against activated p53. Nutlin-3 has a structure mimicking a p53 peptide aptamer, binds to the p53-binding pocket of MDM2, and competitively inhibits p53 binding. **b,** Nutlin-3 rarely affects the cell proliferation of the co-transductant of PB1$_N$- and PA-fused chimera. On the other hand, the p53–MDM2 interaction-dependent cell growth is gradually attenuated in a Nutlin-3-dependent manner. The half maximal inhibitory concentration (IC$_{50}$) is defined as the concentration at which a 50% reduction in relative viable cell density occurred in KIPPIS (3.3±0.2 μM). A cell proliferation assay was performed for determining whether Nutlin-3 disrupt peptide aptamer–polypeptide interaction with 100 nM helper ligand and with serially diluted Nutlin-3 (2-fold dilutions at 11 times; 0.0195–20 nM). The initial cell density was 1×10^5 cells/ml. The viable cell densities were measured after 72 h. The vertical axis represents viable cell densities relative to those without Nutlin-3, which are indicated as mean ± SD (n=3, biological replicates).

(a)

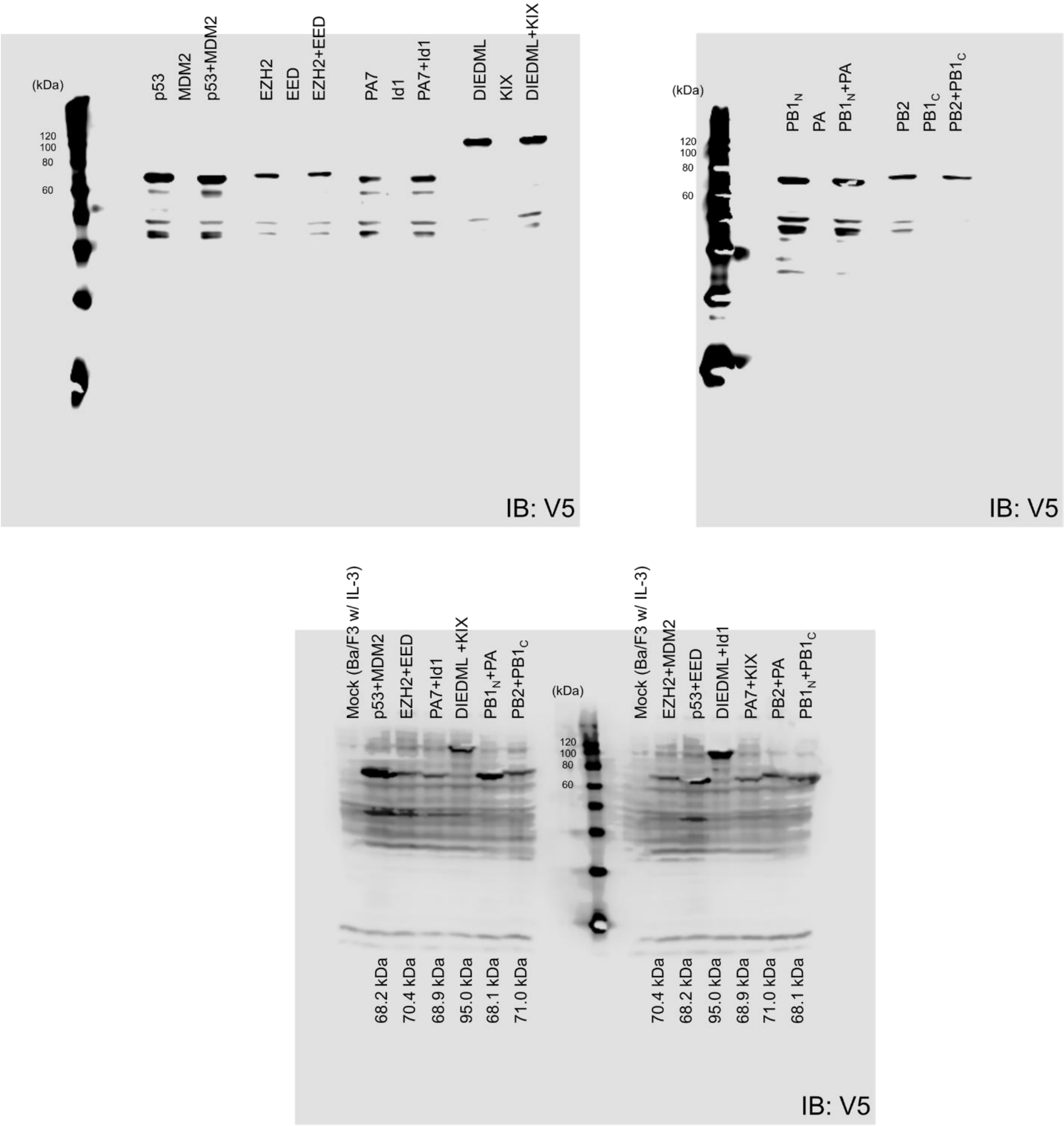

(kDa)
120
100
80
60
p53
MDM2
p53+MDM2
EZH2
EED
EZH2+EED
PA7
Id1
PA7+Id1
DIEDML
KIX
DIEDML+KIX
IB: V5
(kDa)
120
100
80
60
PB1$_N$
PA
PB1$_N$+PA
PB2
PB1$_C$
PB2+PB1$_C$
IB: V5
Mock (Ba/F3 w/ IL-3)
p53+MDM2
EZH2+EED
PA7+Id1
DIEDML +KIX
PB1$_N$+PA
PB2+PB1$_C$
(kDa)
120
100
80
60
Mock (Ba/F3 w/ IL-3)
EZH2+MDM2
p53+EED
DIEDML+Id1
PA7+KIX
PB2+PA
PB1$_N$+PB1$_C$
68.2 kDa
70.4 kDa
68.9 kDa
95.0 kDa
68.1 kDa
71.0 kDa
70.4 kDa
68.2 kDa
95.0 kDa
68.9 kDa
71.0 kDa
68.1 kDa
IB: V5

(b)

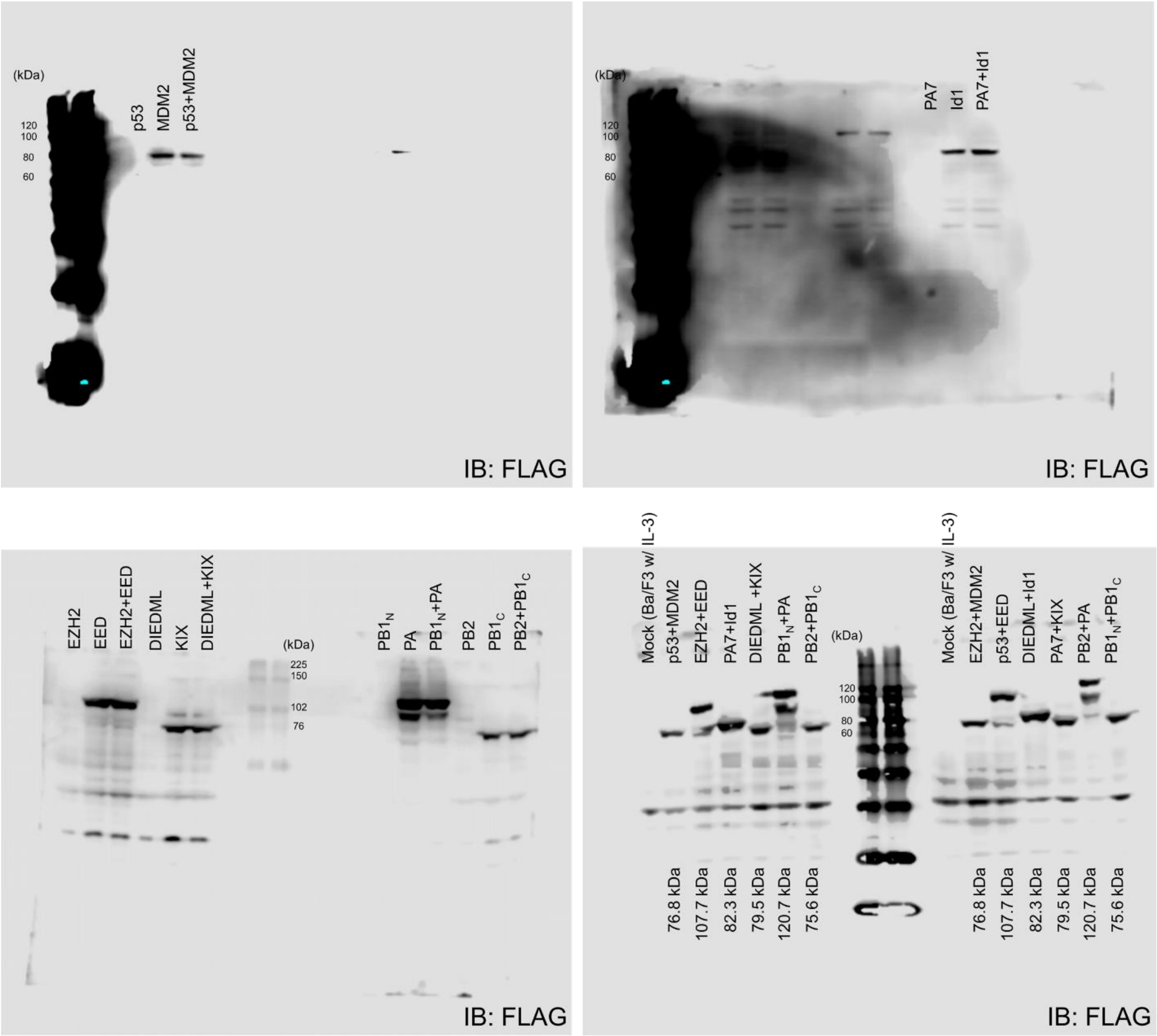
(kDa)
120
100
80
60
p53
MDM2
p53+MDM2
IB: FLAG
(kDa)
120
100
80
60
PA7
Id1
PA7+Id1
IB: FLAG
EZH2
EED
EZH2+EED
DIEDML
KIX
DIEDML+KIX
(kDa)
225
150
102
76
$PB1_N$
PA
$PB1_N$+PA
PB2
$PB1_C$
PB2+$PB1_C$
IB: FLAG
Mock (Ba/F3 w/ IL-3)
p53+MDM2
EZH2+EED
PA7+Id1
DIEDML +KIX
$PB1_N$+PA
PB2+$PB1_C$
(kDa)
120
100
80
60
Mock (Ba/F3 w/ IL-3)
EZH2+MDM2
p53+EED
DIEDML+Id1
PA7+KIX
PB2+PA
$PB1_N$+$PB1_C$
76.8 kDa
107.7 kDa
82.3 kDa
79.5 kDa
120.7 kDa
75.6 kDa
76.8 kDa
107.7 kDa
82.3 kDa
79.5 kDa
120.7 kDa
75.6 kDa
IB: FLAG

(c)

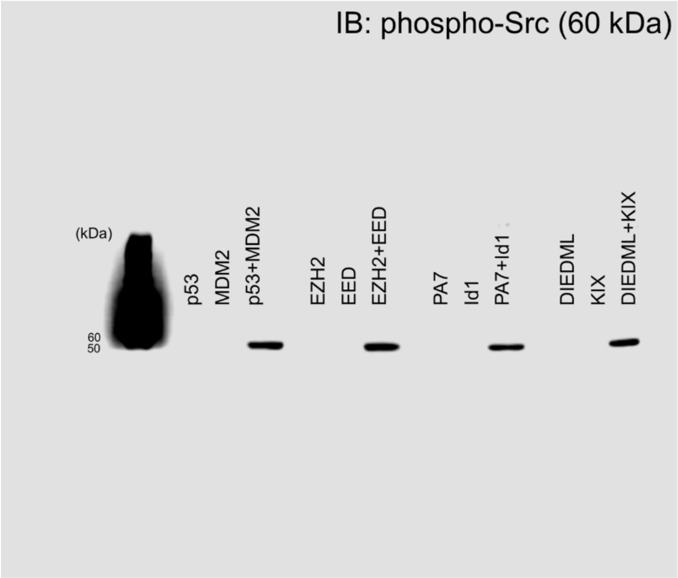
IB: phospho-Src (60 kDa)
(kDa)
60
50
p53
MDM2
p53+MDM2
EZH2
EED
EZH2+EED
PA7
Id1
PA7+Id1
DIEDML
KIX
DIEDML+KIX

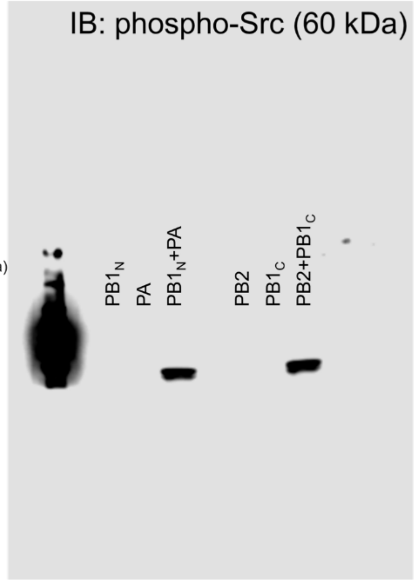
IB: phospho-Src (60 kDa)
(kDa)
60
50
PB1N
PA
PB1N+PA
PB2
PB1C
PB2+PB1C

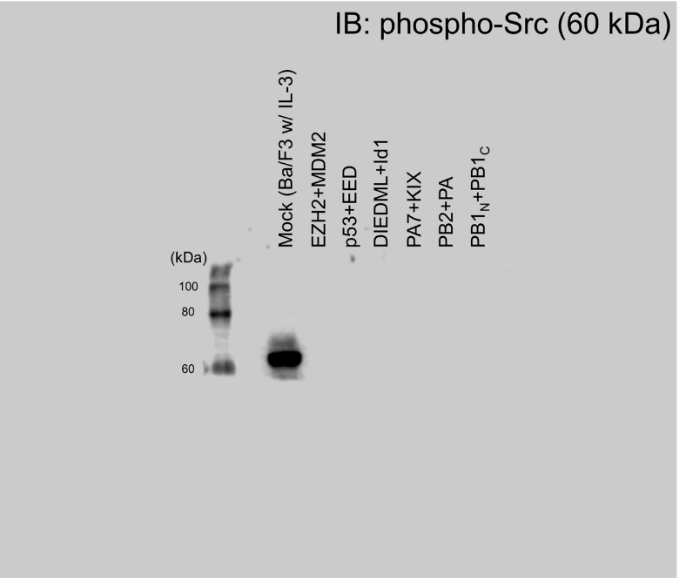
IB: phospho-Src (60 kDa)
(kDa)
100
80
60
Mock (Ba/F3 w/ IL-3)
EZH2+MDM2
p53+EED
DIEDML+Id1
PA7+KIX
PB2+PA
PB1N+PB1C

(d)

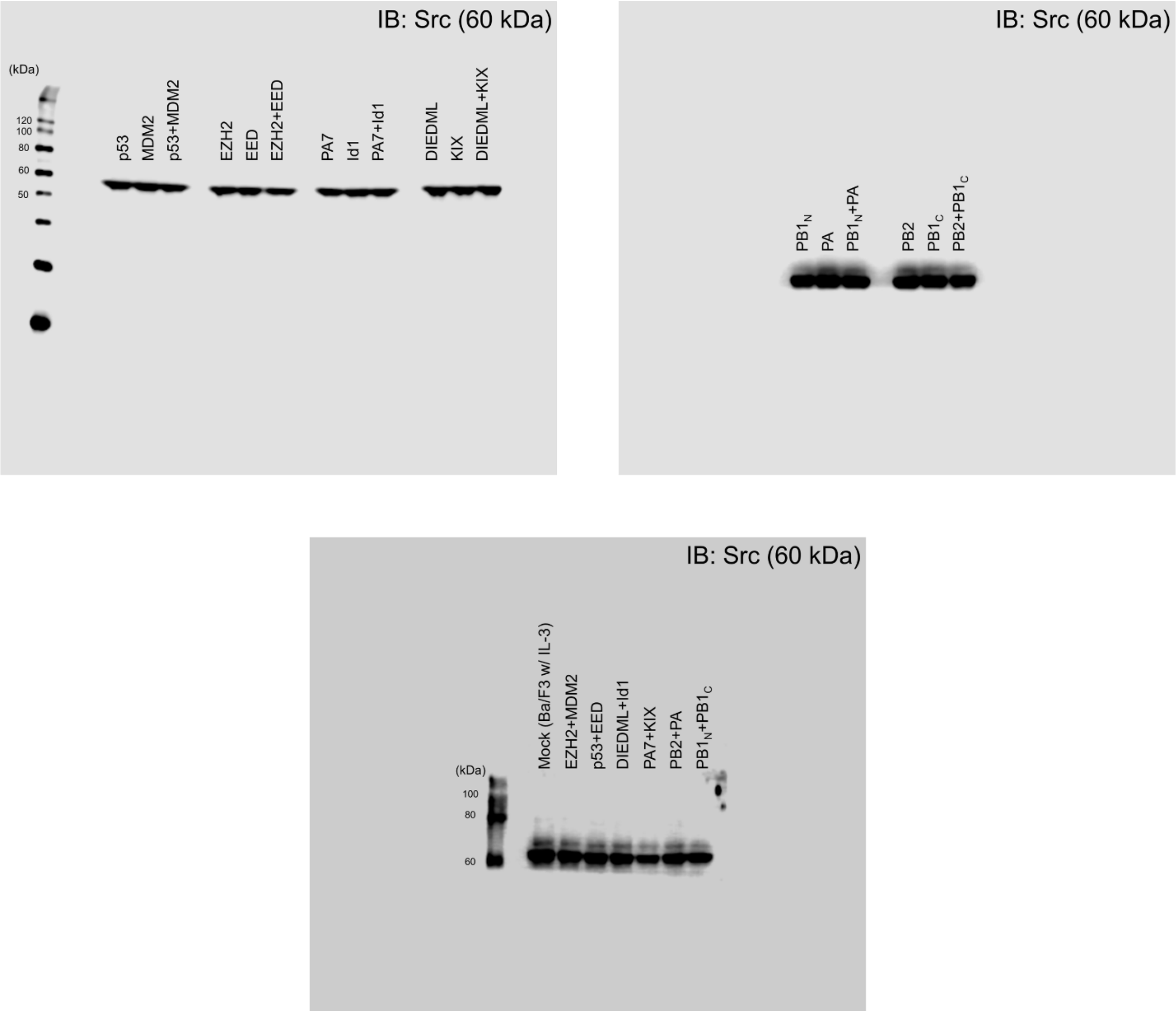
IB: Src (60 kDa)
(kDa)
120
100
80
60
50
p53
MDM2
p53+MDM2
EZH2
EED
EZH2+EED
PA7
Id1
PA7+Id1
DIEDML
KIX
DIEDML+KIX
IB: Src (60 kDa)
$PB1_N$
PA
$PB1_N$+PA
PB2
$PB1_C$
PB2+$PB1_C$
IB: Src (60 kDa)
(kDa)
100
80
60
Mock (Ba/F3 w/ IL-3)
EZH2+MDM2
p53+EED
DIEDML+Id1
PA7+KIX
PB2+PA
$PB1_N$+$PB1_C$

(e)

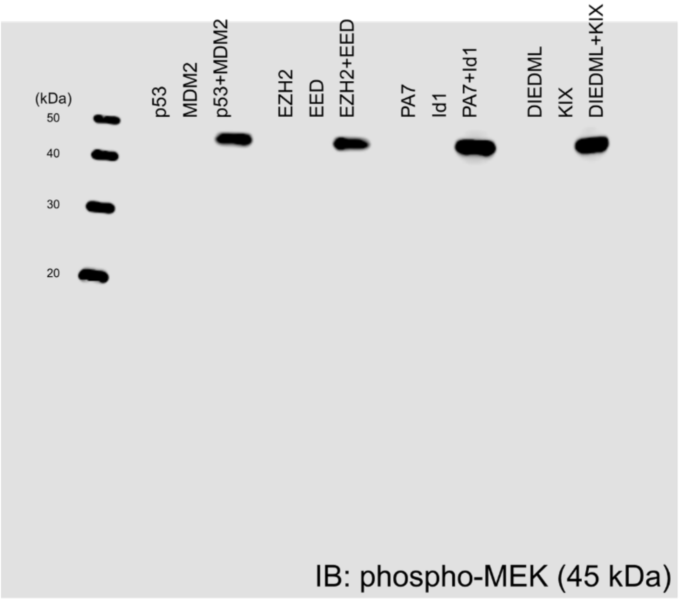

(kDa)
50
40
30
20
p53
MDM2
p53+MDM2
EZH2
EED
EZH2+EED
PA7
Id1
PA7+Id1
DIEDML
KIX
DIEDML+KIX
IB: phospho-MEK (45 kDa)

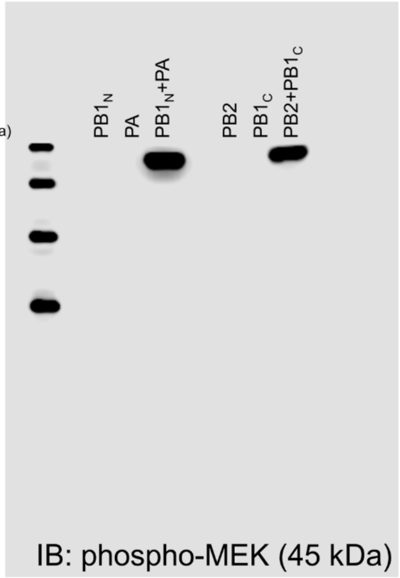

(kDa)
50
40
30
20
PB1N
PA
PB1N+PA
PB2
PB1C
PB2+PB1C
IB: phospho-MEK (45 kDa)

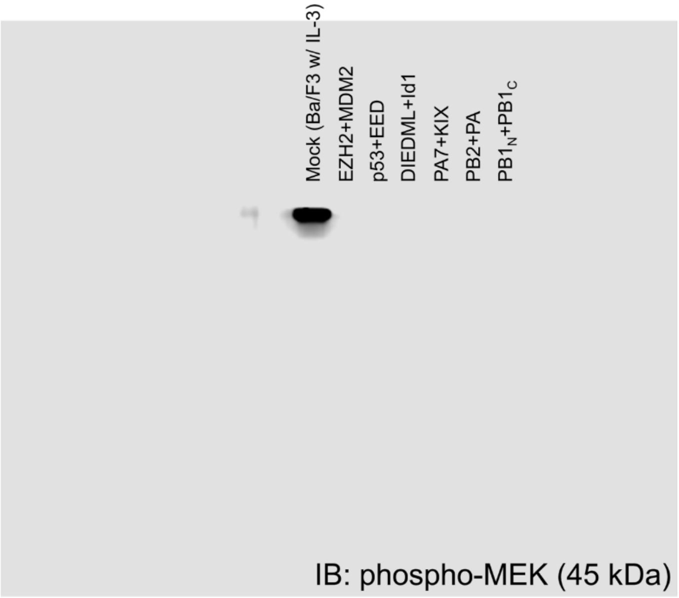

Mock (Ba/F3 w/ IL-3)
EZH2+MDM2
p53+EED
DIEDML+Id1
PA7+KIX
PB2+PA
PB1N+PB1C
IB: phospho-MEK (45 kDa)

(f)

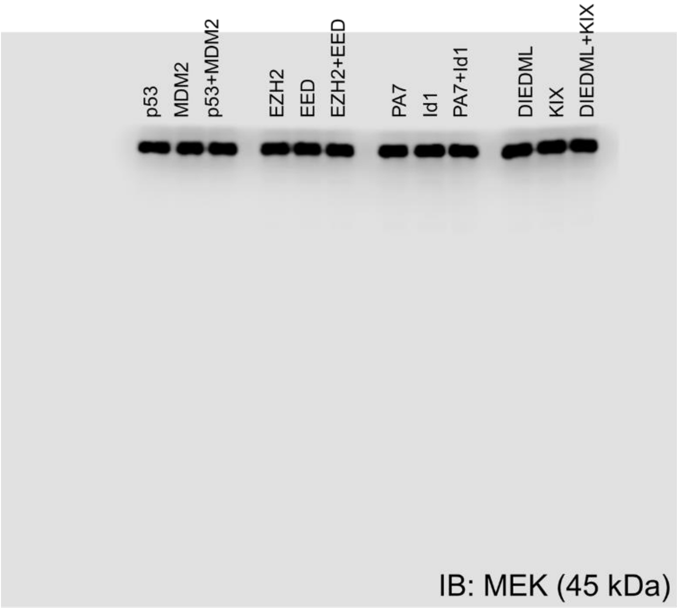
p53
MDM2
p53+MDM2
EZH2
EED
EZH2+EED
PA7
Id1
PA7+Id1
DIEDML
KIX
DIEDML+KIX
IB: MEK (45 kDa)

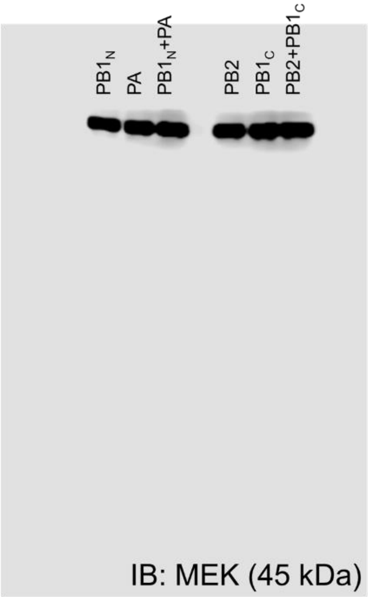
PB1N
PA
PB1N+PA
PB2
PB1C
PB2+PB1C
IB: MEK (45 kDa)

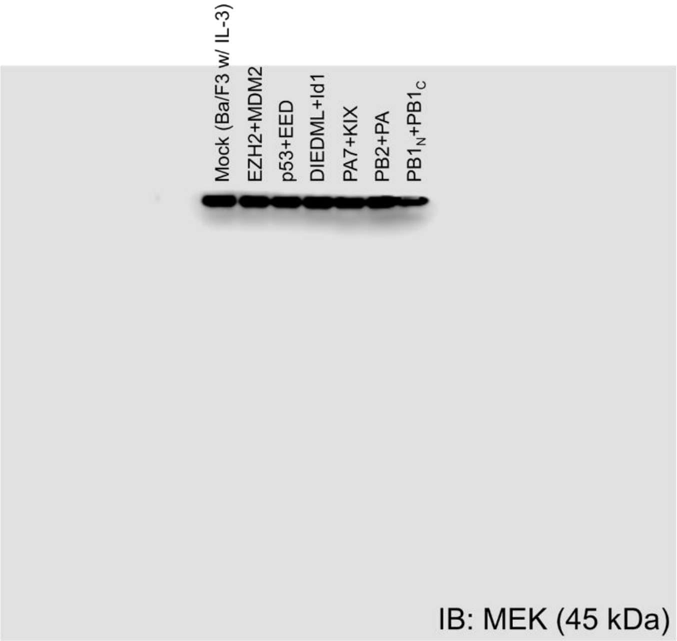
Mock (Ba/F3 w/ IL-3)
EZH2+MDM2
p53+EED
DIEDML+Id1
PA7+KIX
PB2+PA
PB1N+PB1C
IB: MEK (45 kDa)

(g)

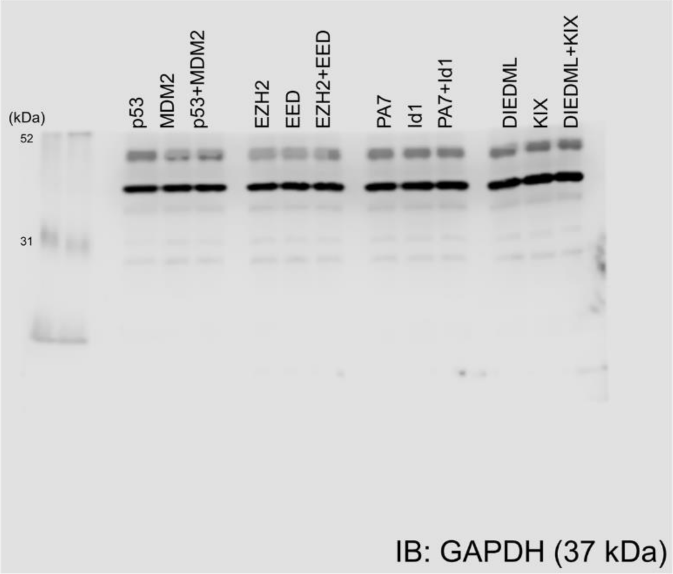
(kDa)
52
31
p53
MDM2
p53+MDM2
EZH2
EED
EZH2+EED
PA7
Id1
PA7+Id1
DIEDML
KIX
DIEDML+KIX
IB: GAPDH (37 kDa)

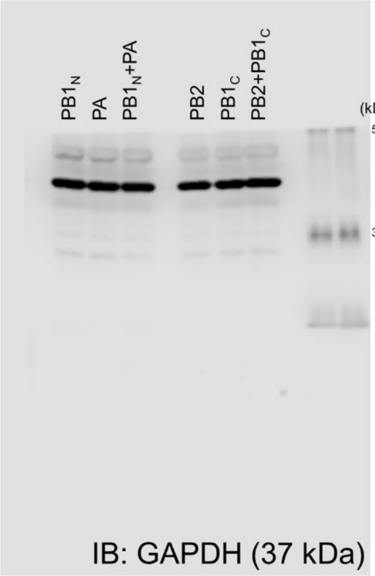
$PB1_N$
PA
$PB1_N$+PA
PB2
$PB1_C$
PB2+$PB1_C$
(kDa)
52
31
IB: GAPDH (37 kDa)

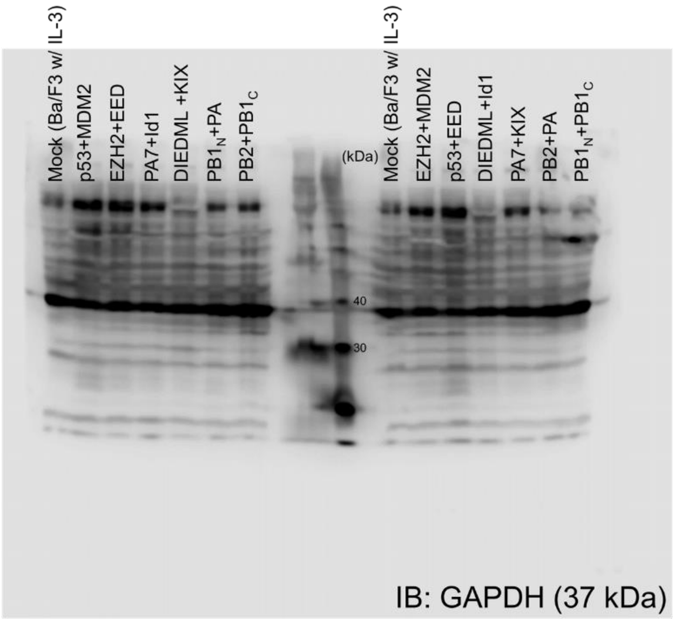
Mock (Ba/F3 w/ IL-3)
p53+MDM2
EZH2+EED
PA7+Id1
DIEDML +KIX
$PB1_N$+PA
PB2+$PB1_C$
(kDa)
40
30
Mock (Ba/F3 w/ IL-3)
EZH2+MDM2
p53+EED
DIEDML+Id1
PA7+KIX
PB2+PA
$PB1_N$+$PB1_C$
IB: GAPDH (37 kDa)

Fig. S5. Full-length blot images. The chimeras expressed in the Ba/F3 transductants are shown as the abbreviated names. The expression levels of the chimeras were determined by immunoblotting for the V5 (a) and FLAG (b) tagged with the peptide aptamer- and polypeptide-fused chimeras, respectively. The expression and phosphorylation levels of endogenous signaling molecules were checked by immunoblotting for the following molecules: phospho-Src (c), Src (d), phospho-MEK (e), and MEK (f). The endogenous protein GAPDH was compared in parallel as a loading control (g). The images were created by an Image Studio software (ver 4.0; https://www.licor.com/bio/image-studio/) associated with a C-DiGit scanner.
